# A Case of Relapsing Polychondritis: Unmasking the Otitis Externa Mimic

**DOI:** 10.7759/cureus.64070

**Published:** 2024-07-08

**Authors:** Vandana Bandari, Ben Hur Aguilar

**Affiliations:** 1 Internal Medicine, Bayhealth Medical Center, Dover, USA

**Keywords:** chondritis, otitis, otitis externa, polychondritis, relapsing polychondritis

## Abstract

Relapsing polychondritis (RPC) is a rare autoimmune condition that often mimics recurrent external otitis. This multisystemic disease primarily affects cartilaginous structures in the body, with the ear pinna being the most commonly impacted. RPC is associated with elevated inflammatory markers and antinuclear antibodies (ANA), and it can lead to chondral destruction. Our case is a 74-year-old Caucasian male with a history of peripheral vascular disease (PVD) who presented to the clinic with recurrent, painful swelling of the right upper ear for 14 days despite multiple antibiotics and nonsteroidal anti-inflammatory drugs (NSAIDs). He had chronic sensorineural hearing loss in the same ear. He was seen multiple times with identical symptoms in the last seven months and was diagnosed with otitis externa. He denied arthritis, fatigue, rash, abrasion, allergies, trauma, or fever. He was prescribed antimicrobials, alternating NSAIDs, and methylprednisolone with temporary relief. He is only on statins and has an unremarkable family history. He was afebrile with normal vital signs. On physical examination, he was not in acute distress and had a normal voice but had a diffusely erythematous, tender, swollen right ear pinna and external canal sparing the lobe. The rest of the physical examination was unremarkable. Laboratory results showed elevated C-reactive protein (CRP) of 100 mg/L (normal range: <3 mg/L) and erythrocyte sedimentation rate (ESR) of 200 mm/hour (normal range: <20 mm/hour). ANA titer is 1:160 with a homogenous pattern, but other autoantibodies were negative. No red flags were noted on the complete blood count (CBC) or comprehensive metabolic panel (CMP), and his rapid plasma reagin (RPR) test was negative.

In this patient, prednisone 60 mg daily was initiated as monotherapy, and rheumatology was also consulted. The patient sought consultation due to recurrent and persistent upper ear infections despite antibiotic treatment and was ultimately diagnosed with a rare medical condition called relapsing polychondritis. Following this treatment, the auricular chondritis improved promptly. The steroid dosage was then slowly tapered and maintained at 10 mg daily to prevent flare-ups. Subsequently, after the initiation of corticosteroids, inflammatory markers trended down to normal levels.

## Introduction

Relapsing polychondritis (RPC) is a rare autoimmune disorder primarily affecting the cartilaginous structures in the body, presenting with systemic manifestations. Despite discovering its first case in 1923, its etiopathogenesis remains inconclusive [1]. The estimated annual incidence is 3.5 instances for every one million people [2]. Up to one-third of the patients exhibited circulating antibodies targeting type II collagen, with the levels of these antibodies correlating with the severity of the disease [3,4]. Although RPC can occur in people of all age groups, it commonly begins in the fifth decade of life, with some studies observing a slight predominance in females. As many as 30% of the patients with relapsing polychondritis might have other associated autoimmune disorders such as systemic vasculitis, systemic lupus erythematosus (SLE), Sjögren's syndrome, rheumatoid arthritis, ankylosing spondylitis, and malignancies.

The diagnosis heavily relies on the clinical presentation, response to treatment, and histological findings. The criteria established by McAdam et al. [5], Michet et al. [6], and Damiani and Levine [7] aid in the diagnostic process. Due to its infrequent incidence, treatment approaches, including corticosteroids, immunomodulatory agents, and surgical interventions, have been developed based on research from previous case reports.

Here, we describe a case involving a 74-year-old male patient exhibiting sensorineural hearing loss and auricular chondritis, which mimicked otitis externa. Despite multiple clinic visits due to recurrent unresolved episodes, the patient was eventually diagnosed with relapsing polychondritis and treated appropriately.

## Case presentation

A 74-year-old Caucasian male patient with a past medical history of peripheral vascular disease (PVD) and right sensorineural hearing loss presented to the outpatient primary provider clinic with complaints of painful swelling of the right ear for 14 days that persisted despite multiple courses of antibiotics, nonsteroidal anti-inflammatory drugs (NSAIDs), and methylprednisolone with only minimal relief. He denies any prior trauma, visits to the water park, allergies, or eczema. His only medication was a statin.

He recounted experiencing similar episodes multiple times throughout the year, previously diagnosed as otitis externa. Treatment with antimicrobials, NSAIDs, and methylprednisolone provided only temporary relief.

**Figure 1:**
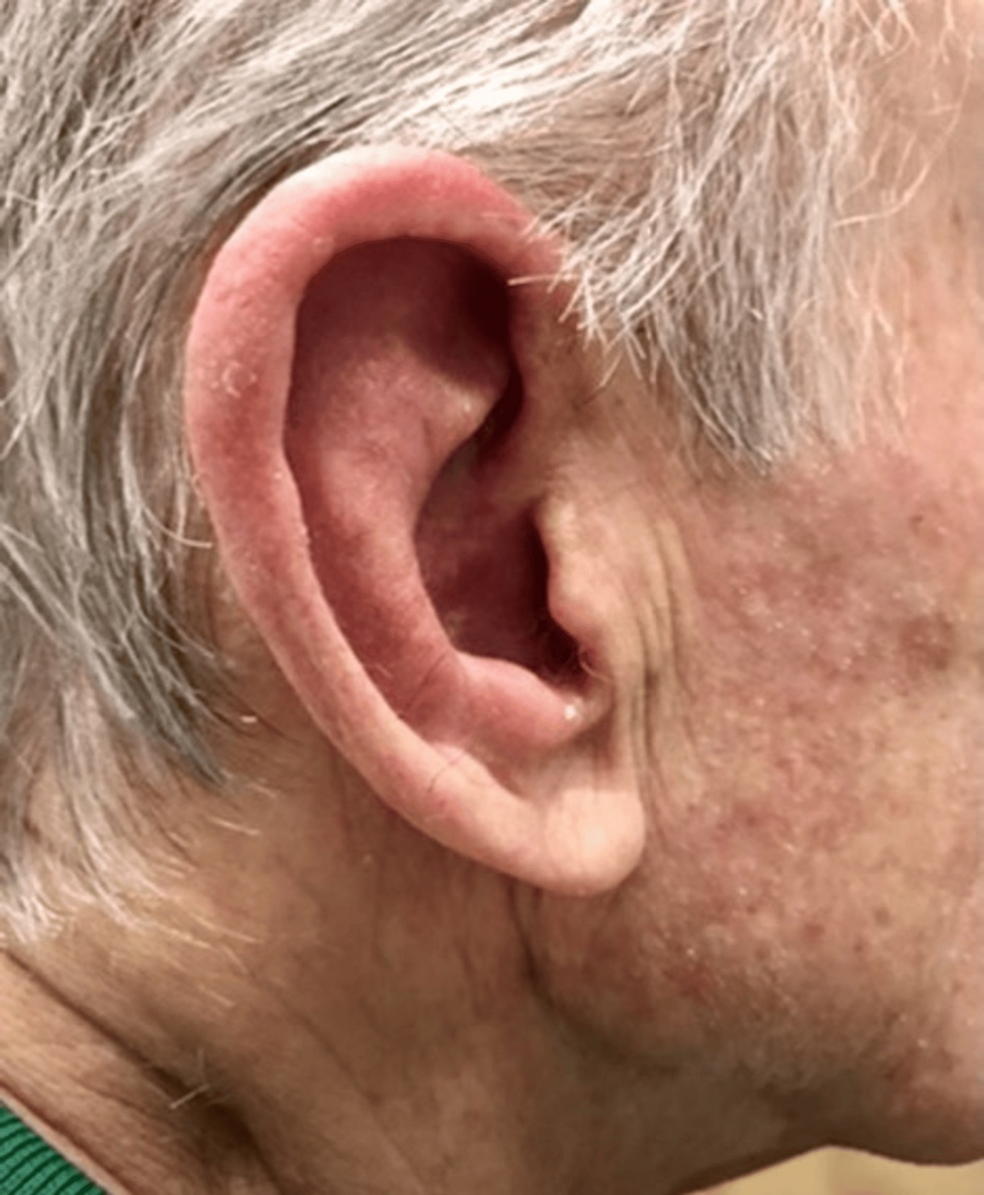
Pathognomonic auricular swelling sparing the ear lobe.

On examination, the patient was hemodynamically stable and afebrile. He exhibited a diffusely erythematous, tender, swollen right ear pinna and external ear canal, sparing the lobe (Figure 1). The examination of his nose, oropharynx, eyes, and left ear was unremarkable.

His blood work (Table 1) was significant for elevated inflammatory markers, including a C-reactive protein (CRP) of 100 mg/L, an erythrocyte sedimentation rate (ESR) of 200 mm/hour, and an antinuclear antibody (ANA) titer of 1:160 with a homogenous pattern but negative extractable nuclear antigen (ENA) and rapid plasma reagin (RPR). The complete blood count (CBC) and metabolic panel were unremarkable. The patient responded positively to steroid treatment, starting with prednisone at 60 mg daily and gradually tapering to a maintenance dose of 10 mg daily to prevent flares. This resulted in the significant resolution of auricular chondritis and the normalization of inflammatory markers.

**Table 1:** Laboratory results of the patient with reference values. CO_2_, carbon dioxide; BUN, blood urea nitrogen; ALT, alanine aminotransferase; AST, aspartate aminotransferase; ALP, alkaline phosphatase; MCV, mean corpuscular volume; MCH, mean corpuscular hemoglobin; MCHC, mean corpuscular hemoglobin concentration; ESR, erythrocyte sedimentation rate; CRP, C-reactive protein; ANA, antinuclear antibody

Laboratory test	Patient's result	Reference value
Serum sodium	137 mmol/L	135-145 mmol/L
Serum potassium	3.8 mmol/L	3.5-5.2 mmol/L
Serum chloride	105 mmol/L	99-109 mmol/L
Serum CO_2_	26 mmol/L	24-35 mmol/L
Serum BUN	7 mg/dL	5-21 mg/dL
Serum creatinine	0.8 mg/dL	0.4-1.1 mg/dL
Serum ALT	11 U/L	10-43 U/L
Serum AST	17 U/L	13-41 U/L
Serum ALP	43 U/L	42-119 U/L
Serum total protein	6.7 g/dL	6.4-8.3 g/dL
Serum albumin	3.9 g/dL	3.5-5.0 g/dL
Serum calcium	8.9 mg/dL	8.3-10.2 mg/dL
Serum magnesium	1.7 mg/dL	1.5-2.5 mg/dL
Serum glucose	85 mg/dL	70-110 mg/dL
Hemoglobin	13.5 g/dL	12.0-16.0 g/dL
Hematocrit	42%	38.0%-47.0%
White blood cells	4.7×10^3^/µL	4.5-11.0×10^3^/µL
Red blood cells	4.8×10^6^/µL	4.20-5. 4×10^6^/µL
MCV	88 fL	81.0-98.0 fL
MCH	29 pg	27.0-32.0 pg
MCHC	33 g/dL	32.0-36.0 g/dL
Platelets	145×10^3^/µL	140-450×10^3^/µL
ESR	200 mm/hour	0-20 mm/hour
CRP	100 mg/L	<0.3 mg/L
ANA	1:160	Less than 1:40

Based on clinical observations and diagnostic criteria by McAdam et al. [5] and Damiani and Levine [7], the patient was diagnosed with relapsing polychondritis, evidenced by auricular chondritis, audiovestibular damage, and a favorable response to steroid therapy.

The patient was ultimately referred to the rheumatology subspecialty clinic for follow-up.

## Discussion

Relapsing polychondritis (RPC) is a challenging condition characterized by immune-mediated inflammation targeting cartilage throughout the body. It often manifests with symptoms in the auricular (ear) and nasal regions, though other cartilaginous structures can also be affected. This inflammatory process can lead to various symptoms and complications, making early diagnosis and management crucial for patients [8].

The etiology of relapsing polychondritis is unknown. Prior studies [3,4] indicate that during the active phase of RPC, 33% of the patients displayed circulating antibodies against type II collagen, with their levels correlating with disease activity. Additional research [9,10] revealed that the antibodies are produced not only against native and denatured type II collagen but also against type IX and XI collagen, which constitute extracellular scaffold within cartilage [11,12]. In recent studies, the prevalence of human leukocyte antigen (HLA)-DR4 in RPC patients was examined, revealing rates of up to 56% within the patient cohort compared to 26% in the healthy control group. Likewise, a significant association was identified between HLA-DR6 positivity and clinical manifestations of RPC, although the precise implications of this finding remain unclear [10]. While RPC can manifest across all age groups, it typically initiates in the fifth decade of life. Generally, studies [5,6] have indicated no notable gender preference, although Trentham and Le [9] noted a slight predominance of females [13]. The estimated annual incidence is 3.5 cases per one million individuals [2].

Up to a third of RPC patients may have a concurrent condition, such as systemic vasculitis, dermatologic or hematologic disorders, or other systemic rheumatic diseases. A growing number of RPC instances have been associated with malignancies, notably myelodysplastic syndrome (MDS), and less commonly solid tumors affecting the bladder, breast, lung, colon, and pancreas, as well as other hematologic malignancies such as lymphoma. These comorbidities might precede RPC, emerge after its diagnosis, or coincide with its onset [13].

The mono- or bilateral inflammation of the outer ear cartilage, known as auricular chondritis, is the predominant characteristic of RPC. It occurs in as many as 90% of the patients as the disease progresses and serves as the initial symptom in 20% of cases as in our patient [5,6]. Episodes of acute inflammation typically resolve on their own within a few days or weeks, only to reappear at irregular intervals. However, repeated flares over time lead to significant damage to the cartilage structure, which is gradually replaced by fibrous tissue. This results in a progressive alteration of the ear's normal shape, with nodular or wartlike appearances. The deformity sometimes resembles the characteristic appearance seen in professional boxers known as the "cauliflower ear."

Up to 46% of the patients with RPC experience some form of hearing loss be it conductive or sensorineural hearing loss. Factors such as the collapse of auricular cartilage, canal edema, the closure of the ear canal leading to middle ear inflammation, or the fixation of the stapedial footplate can lead to conductive hearing loss, whereas inflammation affecting vestibular structures or vasculitis affecting the internal auditory artery can lead to sensorineural hearing loss, as could be the case in our patient. Additionally, RPC patients may experience otitis externa, chronic inflammation of the eardrum (myringitis), and persistent ringing in the ears (tinnitus) [13].

Arthropathy is the second most common presenting symptom in patients with RPC. It occurs during the disease course in around 50%-80% of the patients but as an initial feature in only 33% of the patients. It manifests as acute, asymmetric, intermittent poly- or oligoarthritis, commonly affecting the metacarpophalangeal and proximal interphalangeal joints and the knees.

Additional clinical manifestations encompass ocular, neurological, cardiovascular, renal, and dermatologic symptoms [5,6,14,15].

Diagnosis

Relapsing polychondritis lacks distinctive clinical, radiological, and histopathological characteristics. Diagnosis relies on a combination of clinical symptoms, supplementary laboratory tests, radiological examinations, and the biopsy of cartilaginous sites.

McAdam et al. outlined the diagnostic criteria stating that RPC can be diagnosed if three or more of the six clinical features (auricular chondritis, nonerosive inflammatory polyarthritis, nasal chondritis, ocular inflammation, respiratory tract chondritis, and audiovestibular damage) are present, without the need for histological confirmation [5]. These criteria were later revised by Damiani and Levine [7], who broadened the diagnostic criteria by including the presence of at least one of McAdam et al.'s [5] criteria and positive histologic confirmation or two of McAdam et al.'s [5] criteria and a positive response to corticosteroid or dapsone treatment [15]. Another adaptation of McAdam et al.'s [5] criteria was proposed by Michet et al. in 1986 [6], stating that the diagnosis of RPC necessitates confirmed inflammation in two of the three cartilages (auricular, nasal, or laryngotracheal) or, alternatively, proven inflammation in one of these cartilages and two additional minor criteria such as hearing loss, ocular inflammation, vestibular dysfunction, or seronegative arthritis [6]. The above-discussed diagnostic criteria are depicted in Table 2.

**Table 2:** Diagnostic criteria for relapsing polychondritis.

Authors	Diagnostic criteria
McAdam et al. [5]	Positive for at least three out of six clinical features: 1) auricular chondritis; 2) nonerosive inflammatory polyarthritis; 3) chondritis of nasal cartilage; 4) ocular inflammation, scleritis/uveitis/conjunctivitis; 5) inflammation of the respiratory tract; and 6) cochlear and/or vestibular damage, conductive/sensorineural hearing loss, tinnitus, and vertigo
Damiani and Levine [7]	At least one out of six features outlined by McAdam et al. [5] plus histological confirmation or two out of six features outlined by McAdam et al. [5] plus positive response to either dapsone or corticosteroids
Michet et al. [6]	Inflammation confirmed in two out of three cartilages (auricular, nasal, or laryngotracheal) or inflammation proven in one of these cartilages, along with two additional minor criteria, such as hearing loss, ocular inflammation, vestibular dysfunction, or seronegative arthritis

Our patient has satisfied the diagnostic criteria per Damiani and Levine [7], with his auricular chondritis and sensorineural hearing loss accounting for two out of the six clinical features outlined by McAdam et al. [5] plus a positive response to treatment with prednisone.

Management

Due to the rarity of RPC, there are only a few clinical trials evaluating treatment options. Pharmacological strategies rely heavily on extensive collections of individual case reports. While treatments have shown effectiveness in alleviating symptoms, none have been demonstrated to alter the disease's natural progression.

Patients presenting with nasal, auricular, and articular chondritis but without visceral complications may be treated with anti-inflammatory drugs, colchicine, or dapsone, although their efficacy is limited [5,6]. Low-dose glucocorticoid therapy is often necessary. For those with an involvement of the large airways, eyes, cardiovascular system, nervous system, or kidneys, initial treatment depends on the severity of the disease. Oral glucocorticoids may suffice for those with mild symptoms. However, individuals with potentially severe manifestations, such as severe laryngeal or tracheobronchial chondritis, sudden sensorineural hearing loss, or systemic vasculitis with poor prognostic indicators, may benefit from methylprednisolone bolus therapy (15 mg/kg/day) combined with an immunosuppressive or immunomodulatory agent as an initial treatment. Commonly utilized immunomodulatory medications include cyclophosphamide, methotrexate, azathioprine, and cyclosporine [4,12,16].

## Conclusions

In conclusion, this case report highlights the challenging diagnosis and management of relapsing polychondritis (RPC), a rare autoimmune condition affecting cartilage throughout the body. This case underscores the importance of considering RPC in patients presenting with auricular symptoms and sensorineural hearing loss. Additionally, it emphasizes the necessity for a multidisciplinary approach involving rheumatology, otolaryngology, and other specialties for optimal management. Further research and clinical trials are warranted to enhance our understanding of RPC and improve treatment strategies for affected individuals.
